# The Role of Hyperuricemia in Cardiac Diseases: Evidence, Controversies, and Therapeutic Strategies

**DOI:** 10.3390/biom14070753

**Published:** 2024-06-25

**Authors:** Yue Zheng, Zhirui Chen, Jinya Yang, Jing Zheng, Xiaorong Shui, Yiguang Yan, Shian Huang, Zheng Liang, Wei Lei, Yuan He

**Affiliations:** 1 Guangdong Provincial Engineering Technology Research Center for Molecular Diagnosis and Innovative Drugs Translation of Cardiopulmonary Vascular Diseases, University Joint Laboratory of Guangdong Province and Macao Region on Molecular Targets and Intervention of Cardiovascular Diseases, Affiliated Hospital of Guangdong Medical University, Zhanjiang 524001, China; zhengyue@gdmu.edu.cn (Y.Z.); czrpork@gdmu.edu.cn (Z.C.); yangjinya@gdmu.edu.cn (J.Y.); yanyg@gdmu.edu.cn (Y.Y.); 2 Laboratory of Cardiovascular Diseases, Affiliated Hospital of Guangdong Medical University, Zhanjiang 524001, China; 3 Department of Obstetrics and Gynecology, University of Wisconsin, Madison, WI 53715, USA; jzheng@wisc.edu; 4 Laboratory of Vascular Surgery, Affiliated Hospital of Guangdong Medical University, Zhanjiang 524001, China; shuixiaor@gdmu.edu.cn; 5 Cardiovascular Medicine Center, Affiliated Hospital of Guangdong Medical University, Zhanjiang 524001, China; huangshian@gdmu.edu.cn (S.H.); liangzheng@gdmu.edu.cn (Z.L.); 6 Precision Medicine Center, Affiliated Hospital of Guangdong Medical University, Zhanjiang 524001, China

**Keywords:** hyperuricemia, myocardial injury, acute myocardial infarction, atrial fibrillation, heart failure

## Abstract

Hyperuricemia (HUA) may lead to myocardial cell damage, thereby promoting the occurrence and adverse outcomes of heart diseases. In this review, we discuss the latest clinical research progress, and explore the impact of HUA on myocardial damage-related diseases such as myocardial infarction, arrhythmias, and heart failure. We also combined recent findings from basic research to analyze potential mechanisms linking HUA with myocardial injury. In different pathological models (such as direct action of high uric acid on myocardial cells or combined with myocardial ischemia-reperfusion model), HUA may cause damage by activating the NOD-like receptor protein 3 inflammasome-induced inflammatory response, interfering with cardiac cell energy metabolism, affecting antioxidant defense systems, and stimulating reactive oxygen species production to enhance the oxidative stress response, ultimately resulting in decreased cardiac function. Additionally, we discuss the impact of lowering uric acid intervention therapy and potential safety issues that may arise. However, as the mechanism underlying HUA-induced myocardial injury is poorly defined, further research is warranted to aid in the development novel therapeutic strategies for HUA-related cardiovascular diseases.

## 1. Introduction

The heart serves as the vital energy generator and primary circulatory organ within an organism. Through its specialized conduction system and rhythmic diastolic contractions, the myocardium propels the circulation of blood, ensuring adequate perfusion to organs and tissues, and thereby sustaining the body’s regular physiological functions and metabolic processes. Multiple factors, such as ischemia-hypoxia [1,2], hyperlipidemia [3], hyperglycaemia [4,5], and viruses [6,7], have the potential to induce myocardial damage, subsequently resulting in myocardial remodeling. This remodeling process entails the substitution of normal myocardial tissue with fibrosis and scarring, thereby compromising cardiac function. In instances of heightened severity, cardiac remodeling may advance to myocardial failure, thereby presenting a substantial peril to the overall well-being of the human organism. Presently, although significant advancements have been achieved in pharmacologic or surgical interventions for the management of cardiac conditions, the primary concern persists in enhancing the mitigation of irreversible myocardial damage or the postponement of heart failure (HF).

Recent studies have provided evidence for the association between hyperuricemia (HUA) and cardiovascular diseases (CVD) [8,9,10]. Epidemiological studies have demonstrated a global increase in the prevalence of patients with HUA or gout [11]. In the United States, approximately 20% of the population suffers from HUA [12], and a study based on a population in Minnesota found that the prevalence of gout has more than doubled, and is associated with an increased incidence of cardiovascular risk factors, making it a major public health challenge in the area [13]. In China, the overall prevalence of HUA in adults is 14%, with a significantly greater prevalence of 24.4% in men than in women (3.6%) [14]. HUA has been found to be correlated with an elevated 10-year risk of atherosclerotic cardiovascular disease [15]. Serum uric acid (SUA) is an independent risk factor for fatal myocardial infarction (MI) [16] and lethal HF [17], as well as a predictor of all-cause mortality and cardiovascular mortality in patients with cardiometabolic disease who are not diagnosed with CVD [18]. The prevalence of HUA is not only increasing but also affecting younger individuals due to improvements in living conditions, alterations in dietary habits, and increasing societal pressures [19]. The monitoring of SUA and the implementation of early intervention and management strategies for HUA may prove to be an efficacious approaches for the prevention and treatment of CVD in the near future.

**Figure 1:**
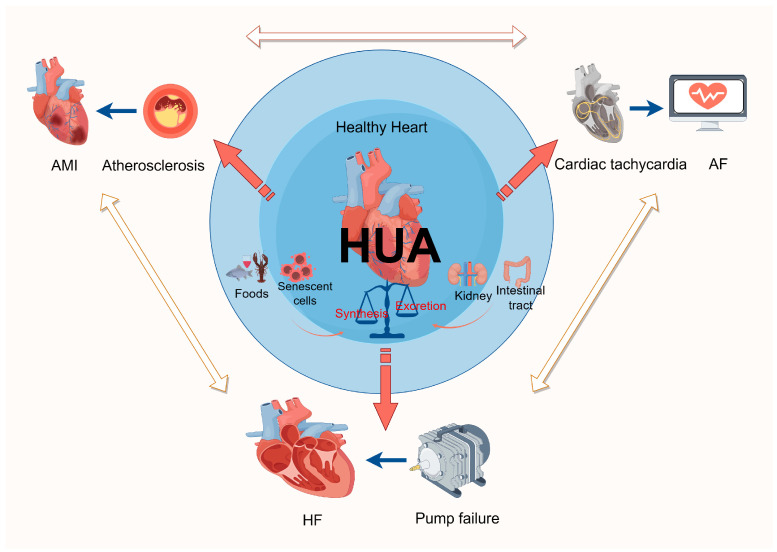
Summary of the association between hyperuricemia and the development of cardiac diseases. AMI, acute myocardial infarction; HUA, hyperuricemia; AF, hyperuricemia; HF, heart failure.

Uric acid (UA) is the final metabolite resulting from the breakdown of purines, with the predominant source of SUA being the degradation of purines derived from senescent cells within the organism, along with a minor contribution from purines obtained through dietary intake. The elimination of UA primarily occurs via renal excretion in the form of urine, and the concentration of UA in the bloodstream is contingent upon the equilibrium between its synthesis and excretion. When the concentration of SUA exceeds the physiologic range, it can be diagnosed as HUA. HUA may lead to myocardial cell damage, thereby promoting the occurrence and adverse outcomes of heart diseases (Figure 1). Therefore, a comprehensive understanding of the role and mechanisms of HUA in myocardial injury is essential for the prevention and management of these diseases.

## 2. Current Status of Clinical Research on HUA and Myocardial Injury-Related Diseases

Several recent clinical data analyses have demonstrated a significant correlation between elevated levels of UA and the incidence of heart diseases [20,21]. Since the mid-20th century, scholars have extensively investigated the association between HUA and coronary artery disease (CAD) [22].

### 2.1. HUA Is Involved in MI Development and Prognosis

MI is characterized by irreversible necrosis of the myocardium, which is primarily attributed to prolonged ischemia and hypoxia of the coronary arteries. This condition is most frequently associated with CAD. Previous research has proposed a potential association between HUA and significant adverse cardiac events in individuals diagnosed with acute myocardial infarction (AMI) [23]. Furthermore, elevated levels of UA have been linked to an increased risk of AMI. The URRAH study conducted in Italy discovered that a specific threshold level of SUA (>5.7 mg/dL) could be identified to differentiate the occurrence of a fatal MI event, even after accounting for various confounding variables. Additionally, a study revealed that high UA levels could serve as a surrogate marker for the severity of CAD in a female population [16]. In multiple prospective cohort studies that enrolled participants without a history of MI and followed them for an extended period, the monitoring of changes in SUA levels and the documented occurrence of cardiovascular disease revealed that earlier exposure to a hyperuricemic environment, at an equivalent level of SUA accumulation, had a greater impact on the development of MI and overall mortality [21]. The dynamic monitoring of the trajectory of SUA changes has the potential to aid in the early identification of subgroups at risk for MI and all-cause mortality [24]. Similarly, in a study conducted by Nakanishi et al. [25], elevated UA levels were independently associated with subclinical left heart failure in a general population sample devoid of significant cardiac ailments. These studies share the common characteristic of including participants without a history of MI or cardiac disease, implying that prolonged exposure to elevated levels of UA may confer an increased risk of future heart disease, particularly MI.

HUA potentially contributes to the advancement of coronary atherosclerotic lesions. A retrospective examination was conducted on patients who were diagnosed with acute coronary syndrome and admitted to an emergency department in a Japanese hospital. Two distinct groups were formed based on an SUA concentration of 6 mg/L, which served as a threshold value. A comparative analysis was conducted on the findings derived from optical coherence tomography scans of coronary arteries before percutaneous coronary intervention (PCI). The investigation unveiled a greater prevalence of plaque rupture, plaque erosions or calcified nodules, and red thrombi among patients with acute coronary syndrome in the high unstable angina group [26]. These occurrences are recognized as the underlying factors of CAD and a frequent contributor to secondary myocardial ischemia and hypoxia. A subsequent examination of the findings obtained from cardiac magnetic resonance imaging conducted during the acute (3–5 days) and follow-up (4–6 months) phases in individuals diagnosed with ST-segment elevation MI revealed that the HUA group exhibited a greater extent of MI in both datasets. Nevertheless, the decrease in infarct size did not significantly differ from that in the normal UA group [27]. The findings of this study demonstrated a statistically significant association between elevated levels of UA and the severity of coronary lesions. These results suggest that UA may play a contributing role in the pathogenesis of myocardial ischemia-reperfusion injury, specifically in the development of coronary atherosclerotic lesions.

The prognosis for recovery and survival becomes less optimistic as the area of myocardial injury increases. Clinical studies have shown that patients with HUA and AMI exhibit an elevated risk of experiencing HF and cardiogenic shock during hospitalization, as well as a diminished likelihood of achieving adequate coronary blood flow recovery following PCI. Furthermore, these patients face a higher probability of short-term mortality and an unfavorable long-term prognosis [28,29,30,31,32]. The independent association between HUA and the prognosis of major adverse cardiovascular events has been observed in both elderly and relatively young populations [33,34]. These findings provide additional evidence indicating that UA not only elevates the risk of myocardial injury but also influences the extent of prognosis and recovery.

Early exposure of the heart to HUA increases the risk of MI and can also contribute further to MI by promoting the development of coronary atherosclerosis, resulting in myocardial damage. Moreover, HUA has a serious impact not only on the recovery and prognosis of MI patients but also on the effectiveness of treatment. Furthermore, the aforementioned studies employed lower SUA thresholds to identify hyperuricemic groups at elevated risk for MI outcomes, which were demonstrably lower than the levels utilized to define HUA in clinical practice [35]. Therefore, regular monitoring of SUA levels is highly important for the early detection of HUA and early management.

### 2.2. HUA Increases the Risk of Atrial Fibrillation (AF)

AF is the most common form of dysrhythmia [36]. HUA has been recognized as an independent risk factor for AF [37]. The risk of AF increases by 15% in men and 35% in women for every 1 mg/dL increase in SUA levels [38]. In a comprehensive analysis of a large cross-sectional study, Kuwabara and colleagues observed significantly higher UA levels in the AF group compared to the non-AF group. Furthermore, the odds ratio for the risk of AF in patients with concurrent HUA, in the absence of renal impairment and chronic underlying diseases, was found to be 3.19 (95% CI, 1.81–5.62) [39]. In a cross-sectional study conducted by Mantovani and colleagues, patients diagnosed with type 2 diabetes mellitus and concurrent HUA were enrolled. The examination of data obtained from 24-h ambulatory electrocardiographic monitoring revealed a significant fourfold increase in the likelihood of paroxysmal atrial fibrillation among individuals with comorbid diabetes mellitus and HUA [40]. Based on a prospective cohort study conducted by Li et al., which involved over 120,000 Chinese patients and spanned 8 years with regular follow-up, biennial UA measurements, and electrocardiographic assessment of AF events, it was concluded that the cross-sectional design’s limitation of only one measurement of SUA did not hinder the identification of an association between elevated SUA levels over time and an increased risk of AF development [41]. These findings indicate a potentially significant impact of HUA on the development of AF.

The presence of HUA has been found to elevate the likelihood of AF in individuals with abnormal glucose metabolism [42]. Sodium-glucose cotransporter 2 (SGLT2) plays a crucial role in regulating blood glucose reabsorption within the proximal renal tubular epithelium. From a clinical perspective, SGLT2 inhibitors have demonstrated efficacy in reducing blood glucose levels in diabetic patients while also exerting an influence on SUA and magnesium levels, thereby mitigating the incidence of AF [36]. In cardiomyocytes exposed to high glucose, SGLT2 expression has been shown to be significantly increased [43]. As a consequence, the aforementioned phenomenon may lead to increased efficiency of glucose transporters and uric acid transporters within cardiomyocytes, consequently causing an increase in intracellular glucose and UA levels. This, in turn, may contribute to an imbalance in ion levels between the intracellular and extracellular environments, irregularities in electrolyte concentrations, and the progression of AF [36]. Furthermore, the transportation of HUA into cardiomyocytes through transporters may induce modifications in the intracellular fluid milieu of cardiomyocytes, ultimately disrupting cardiac electrophysiology.

### 2.3. HUA Is Associated with the Onset and Outcome of HF

SUA levels have been proposed as a potential indicator for predicting the occurrence of HF during hospitalization in patients with AMI [28]. In HF patients, not only was an interaction observed between SUA levels and ventricular function [44,45], but it was also associated with increased mortality and prognostic outcomes. Huang et al. conducted a study on patients who were admitted to the hospital due to acute HF and reported a positive correlation between the quartile distribution of SUA levels and the risk of mortality [46]. A study conducted on the metabolic exercise cardio-renal inde database revealed that elevated levels of UA in individuals with moderate-to-severe chronic HF, characterized by reduced ejection fraction and cardiac function in class I and II, were indicative of a more unfavorable prognosis [47]. In a subsequent examination of the RELAX trial, HF patients exhibiting elevated SUA levels demonstrated heightened comorbidities and diminished performance in cardiopulmonary exercise testing in comparison to patients without SUA elevation. After accounting for potential confounding variables, elevated SUA levels in HF patients were found to be associated with the serum biomarkers N-terminal pro-BNP and high-sensitivity troponin I, which are indicative of myocardial injury [48]. The results of this study indicate a correlation between increased SUA levels and myocardial injury.

The administration of high-dose diuretics and insufficient renal perfusion in patients with advanced HF can lead to elevated levels of UA. As cardiac function deteriorates and renal function declines, the predictive significance of SUA in HF patients diminishes [46,47]. However, according to the findings of Wu et al., after conducting a 4-year follow-up study on an elderly asymptomatic group with HUA while excluding individuals with preexisting cardiovascular, metabolic, and chronic renal conditions, it was observed that the likelihood of experiencing HF events was 2.34 times greater in the HUA group. This suggests that UA may serve as a potentially valuable biomarker for predicting the onset of congestive HF in elderly patients without any coexisting medical conditions [49]. The prognostic value for HF patients is notably enhanced by the combination of UA with other markers, including pulse pressure and N-terminal pro-BNP [50,51]. HUA interferes with cardiomyocyte function, accelerating the progression of HF and leading to a poor prognosis. Although there is presently no conclusive experimental evidence indicating a direct correlation between HUA and the progression of HF, examination of clinical data reveals that lowering UA levels results in reduced all-cause mortality [52] and enhanced vascular endothelial function in patients with HF [53]. Consequently, comprehending the association between UA and heart disease holds promising clinical advantages, which can be attained through additional future investigations.

### 2.4. Controversies

The association between HUA and cardiometabolic comorbidities is a topic of ongoing debate. Sandoval-Plata et al. [54] conducted a case-control study utilizing data from the UK Biobank and revealed that the relationships between gout and cardiovascular comorbidities, diabetes mellitus, and hypercholesterolemia remained significant regardless of SUA level. Furthermore, the absence of a causal relationship for soluble SUA is also evident in Mendelian randomized studies and randomized controlled trials [55]. The contentious findings can be ascribed to various factors, including the researchers’ choice of research design, methodologies, sample sizes, and the inevitable presence of confounding variables in experimental investigations.

Alternatively, HUA can be categorized into asymptomatic HUA and gout based on the presence or absence of clinical symptoms. The emergence of gout signifies an acute inflammatory reaction within the body, as urate typically constitutes the primary form of UA in the human bloodstream. In the context of a hyperuricemic environment, the formation of monosodium urate (MSU) crystals occurs within the joints and extra-articular regions, thereby inciting inflammation and stimulating episodes of gout. Through both in vivo case-control investigations and in vitro modeling studies [56], scholars have demonstrated that dual-energy computed tomography can detect MSU crystals in the coronary plaques of individuals suffering from gout. Moreover, patients with gout displayed elevated MSU deposition and plaque calcification in their coronary arteries and vascular system, as evidenced by previous studies [57,58]. These findings imply that in the presence of a prolonged hyperuricemic environment, particularly when gout symptoms manifest, the persistent accumulation and precipitation of urate crystals lead to inflammation and damage, ultimately fostering the development of a localized chronic inflammatory microenvironment within the coronary arteries. Elevated UA levels above the physiologic range accelerate the progression of coronary atherosclerosis through a persistent proinflammatory response, ultimately leading to myocardial hypoxia and even MI. When the UA concentration is oversaturated with urate crystals precipitating and accumulating, the inflammatory response is stable; thus the link between gout and cardiovascular comorbidities is independent of the blood UA level. Nevertheless, it remains uncertain whether urate deposition directly within cardiomyocytes causes damage to the myocardium. Investigating this matter in future research would be valuable for enhancing our understanding of the impact of HUA on the myocardium.

## 3. Potential Mechanisms of HUA in Myocardial Injury

### 3.1. Direct Effects of UA on the Myocardium

In the last ten years, a majority of researchers have either exposed cells to high levels of UA or created animal models of HUA to investigate the impact on cardiac and other organ tissues. Presently, the prevailing body of research concurs that HUA has a direct influence on the structural integrity and normal functioning of cells and tissues. For instance, in rats, HUA has been found to induce endothelial damage [59,60], increase susceptibility to AF [61], promote atherosclerosis in mice [62], and impair left heart function [63]. Compared with those of control subjects, histological examination of heart sections obtained from rats and mice with HUA revealed pathological alterations in myocardial structure, including disturbances, exudation, and fibrosis [63,64]. Administration of high concentrations of UA in treatment leads to a decrease in the calcium affinity of troponin within cardiomyocytes, consequently impacting cell shortening and ultimately diminishing cardiomyocyte function [65]. Hence, it is imperative to acknowledge the adverse impacts of elevated levels of UA on the structure and functionality of cardiomyocytes.

#### 3.1.1. Inflammasome Activation

**Figure 2:**
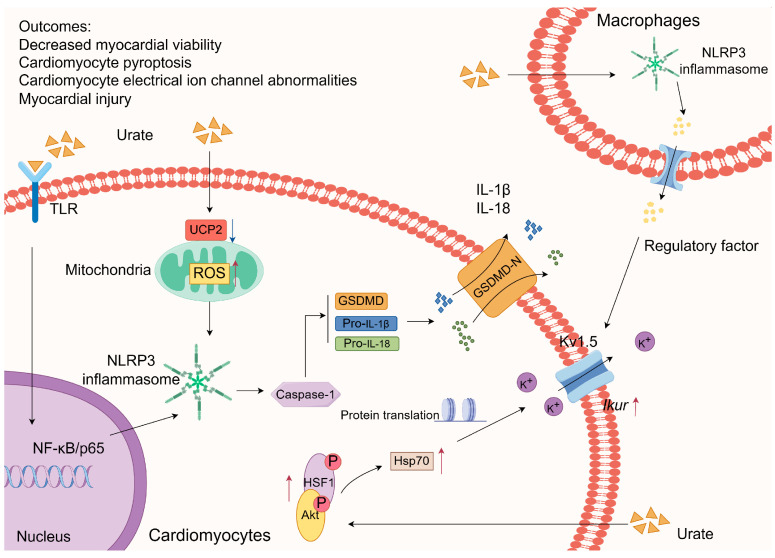
Hyperuricemia mediates myocardial injury by activating the NLRP3 inflammasome and inducing Kv1.5 protein expression. ROS, reactive oxygen species; UCP2, uncoupling protein 2; TLR, toll-like receptor; NLRP3, nod-like receptor family pyrin domain-containing 3; NF-κB, nuclear factorκB; GSDMD, gasdermin D; IL, interleukin; Hsp70, heat shock protein 70; Kv1.5, voltage-gated potassium channel subfamily 1 number 5; Akt, protein kinase B.

According to a previous study, soluble UA enhances the expression of NOD-like receptor protein 3 (NLRP3) by elevating Toll-like receptor 6 (TLR6) levels and activating the NF-κB/p65 pathway as an initial signal for NLRP3 inflammasome activation. Additionally, soluble UA can induce the production of reactive oxygen species (ROS), which is contingent upon the suppression of mitochondrial uncoupling protein 2 (UCP2) expression [66]. This ROS production serves as a secondary signal for NLRP3 inflammasome activation, ultimately leading to myocardial cell injury. Furthermore, the authors observed an improvement in myocardial injury and a decrease in left ventricular remodeling in rats with HUA through the knockdown of TLR6. Additionally, Wu et al. confirmed that HUA inhibits the viability of cardiomyocytes by activating NLRP3 and promoting the production of ROS [67]. The activation of NLRP3 induces the maturation of caspase-1, subsequently triggering the activation of downstream IL-1β and IL-18, as well as the release of the active N-terminal domain of gasdermin D (GSDMD). GSDMD has the ability to permeate the plasma membrane, forming a channel that facilitates the unrestricted movement of ions, leading to cellular swelling and lysis. This process also results in the release of inflammatory factors in the surrounding area, thereby amplifying the inflammatory response through pyroptosis [68]. Li et al. [69] observed activation of the NLRP3 inflammasome and the augmentation of voltage-gated potassium channel subfamily 1 number 5 (Kv1.5) channel currents in HL-1 cells. This effect was achieved by exposing HL-1 cells to supernatants obtained from mouse macrophages treated with MSU and lipopolysaccharide, indicating the potential role of MSU in stimulating the NLRP3 inflammasome in macrophages. Consequently, the secretion of regulatory factors from these activated macrophages may influence neighboring atrial myocytes, resulting in arrhythmias triggered by abnormal Kv1.5 channel currents. Furthermore, a separate investigation demonstrated that UA can augment the expression of heat shock protein 70 (Hsp70) through the facilitation of protein kinase B (Akt)/heat shock factor 1 (HSF1) phosphorylation. Hsp70 hinders protein misfolding and aggregation, stabilizes the expression of Kv1.5 proteins, and enhances channel currents during periods of stress [70]. Therefore, the effect of HUA on cardiomyocytes might be modulated by the activation of the NLRP3 inflammasome and the consequent induction of aberrant Kv1.5 protein expression (Figure 2).

At a physiological pH, urate MSU is the predominant form of UA [71]. When the concentration of UA exceeds saturation in the heart, it can precipitate as urate crystals, which are recognized as endogenous damage-associated molecular patterns by the NLRP3 inflammasome, thereby initiating an inflammatory response program. This finding implies that elevated levels of UA contribute to cardiomyocyte damage through the augmentation of the inflammatory response. This finding implies that elevated UA levels may contribute to cardiomyocyte injury through the modulation of the inflammatory response, with the NLRP3 inflammasome emerging as a key mediator in this process. In recent years, significant advancements have been made in investigating the role of hyperuric acid in mediating organ and tissue injury through the activation of the NLRP3 inflammasome. These conditions include gouty episodes [72], hyperuricemic kidney disease [73,74], nonalcoholic fatty liver disease [75], and intestinal barrier dysfunction [76]. Further comprehensive research, combining both basic and clinical approaches, is needed to determine the potential for mitigating hyperuric acid-induced inflammatory injury in the myocardium or other tissues by targeting the NLRP3 inflammasome.

#### 3.1.2. Interference with Energy Metabolism

**Figure 3:**
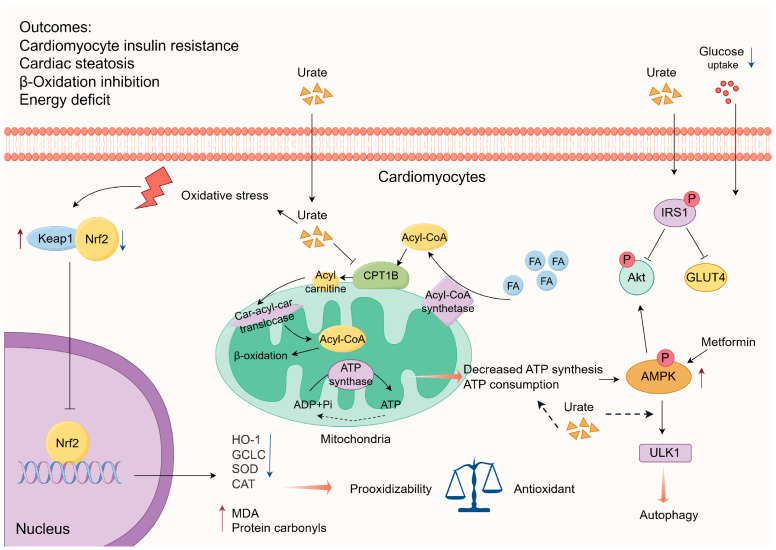
Hyperuricemia disrupts myocardial energy metabolism and impairs the antioxidant system. Car-acyl-car translocase, carnitine-acylcarnitine translocase; ADP, adenosine diphosphate; ATP, adenosine triphosphate; CPT1B, carnitine palmitoyltransferase 1B; Keap1, kelch-like ech associated protein 1; Nrf2, nuclear factor erythroid 2-related factor 2; HO-1, heme oxygenase 1; GCLC, glutamate-cysteine ligase catalytic; MDA, malondialdehyde; SOD, superoxide dismutase; CAT, catalase; IRSI, insulin receptor substrate; Akt, protein kinase B; GLUT4, glucose transporter type 4; AMPK, adenosine 5′-monophosphate (AMP)-activated protein kinase; ULK1, unc-51 like kinase 1.

The maintenance of stable cardiac energy metabolism is essential for the proper functioning of bodily activities. Recent studies have demonstrated that high levels of UA diminish the uptake of glucose by cardiomyocytes through the activation of insulin receptor substrate (IRS1) phosphorylation, subsequently inhibiting Akt phosphorylation and glucose transporter type 4 (GLUT4) translocation [77]. Conversely, metformin counteracts UA-induced insulin resistance in cardiomyocytes by elevating the phosphorylation of adenosine 5′-monophosphate (AMP)-activated protein kinase (AMPK)/Akt [77]. Yu et al. [78] have posited the potential of soluble UA to elicit insulin resistance in the cardiac or cardiomyocyte context. AMPK, a pivotal molecule in the modulation of biological energy metabolism, can be activated by ATP depletion or reduced synthesis, leading to an elevation in AMPK phosphorylation levels [79]. A study demonstrated that HUA stimulates autophagy via the AMPK-ULK1 (unc-51 like kinase 1) pathway, leading to the development of cardiac hypertrophy. The researcher noted a potential association between ATP depletion in cardiomyocytes and the activation of AMPK induced by HUA, although no experimental evidence was presented in the article [80]. Hence, the depletion of the energy supply has adverse effects on the normal functioning and structural stability of cardiomyocytes. Nevertheless, additional research is needed to elucidate the exact underlying mechanism involved. High levels of UA can disrupt myocardial function through interference with energy metabolism, wherein AMPK assumes a pivotal role (Figure 3).

According to research conducted by Yang et al. [81], the study revealed a correlation between cardiomyocyte injury induced by HUA and the accumulation of cytoplasmic lipids. Additionally, the stimulation of UA was found to inhibit the activity of carnitine palmitoyltransferase 1B (CPT1B) on the outer membrane of mitochondria, consequently impeding the transportation of fatty acids (FA) into the mitochondrial matrix. This inhibition ultimately affects the process of fatty acid β-oxidation in the myocardium [81]. In a murine model of hyperuricemic cardiomyopathy, cardiac lipidomics analysis revealed an increase in glycerolipid levels within the myocardium. However, the administration of leucovorin, a pharmacological agent known for its ability to enhance fatty acid β-oxidation, mitigated this effect and attenuated left ventricular hypertrophy [81]. Moreover, research has demonstrated that the chronic consumption of high-fat diets in female Wistar rats results in glucose metabolism abnormalities and cardiac tissue impairment via UA-dependent pathways. This is due to the elevation of body UA levels induced by chronic high-fat diets, which subsequently triggers chronic inflammatory reactions and oxidative stress, ultimately leading to cardiac tissue damage. However, the administration of sodium butyrate has been shown to mitigate these detrimental effects [64]. These studies demonstrate that chronic consumption of high-fat diets can lead to metabolic abnormalities, specifically in the expression of markers such as SUA. Furthermore, the presence of HUA may subsequently impact lipid metabolism abnormalities, resulting in a detrimental metabolic cycle that ultimately impairs long-term myocardial energy metabolism and leads to inevitable myocardial injury (Figure 3).

#### 3.1.3. Weakened Antioxidative Capacity

According to a recent study, it has been observed that optimal levels of UA offer a safeguard against ischemic ECG changes, as evidenced by electrocardiogram [82]. In vitro experiments in H9C2 cells, in which oxidative damage was induced using hydrogen peroxide (H_2_O_2_), demonstrated that UA stimulation led to the upregulation of the expression of nuclear factor erythroid 2-related factor 2 (Nrf2) and its downstream proteins heme oxygenase 1 (HO-1) and glutamate-cysteine ligase catalytic (GCLC) while concurrently downregulating the expression of kelch-like ech associated protein 1 (Keap1) [82]. UA possesses both antioxidant and pro-oxidant attributes, displaying a pro-oxidant inclination when its concentration deviates from the established norm. To illustrate, in an in vitro model employing H_2_O_2_-treated chicken embryo cardiomyocytes to simulate oxidative damage, UA concentrations within the physiological range demonstrated the capacity to safeguard against H_2_O_2_-induced oxidative damage by impeding the inhibition of Nrf2 pathway. The surpassing of the physiological range led to an increase in the concentrations of malondialdehyde (MDA) and protein carbonyls, a decrease in the activities of superoxide dismutase (SOD) and catalase (CAT), and a downregulation in the protein expression of Nrf2 [83]. In the context of a study involving chicken embryo cardiomyocytes treated with high glucose to induce acute oxidative injury, it was consistently observed that high concentrations of UA treatment resulted in a down-regulation of Nrf2 [84]. Nrf2, a crucial transcription factor responsible for regulating antioxidant stress, holds significant importance in the body’s antioxidant response [85]. Hence, in the presence of a hyperuricemic environment, the antioxidant capacity of cardiomyocytes may be diminished, leading to an imbalance and disruption of Nrf2 expression. Consequently, this phenomenon exacerbates oxidative stress and renders the myocardium more vulnerable to injury (Figure 3). However, the treatment of human macrophages with UA (15 mg/dL) notably elevated the expression of Nrf2 and HO-1 [86]. It is important to note that this experimental concentration exceeds the normal range of UA concentrations in humans by more than twofold, potentially attributable to variations in species or cells, as well as the inherent limitation of in vitro experiments in accurately replicating in vivo conditions.

### 3.2. Myocardial Ischemia-Reperfusion Injury (MIRI) Model

#### 3.2.1. Xanthine Oxidase (XO)-Mediated ROS Production

**Figure 4:**
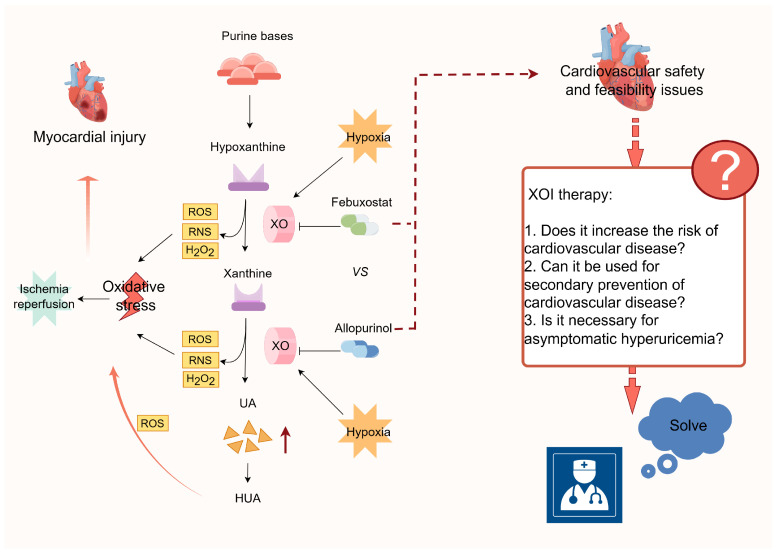
Intervention with xanthine oxidase in cardiovascular disease and issues of cardiovascular safety and feasibility. XO, xanthine oxidase; ROS, reactive oxygen species; RNS, reactive nitrogen species; H_2_O_2_, hydrogen peroxide; UA, uric acid; HUA, hyperuricemia.

Xanthine oxidase inhibitors (XOI) demonstrated a mitigating effect on oxidative damage in a rat MIRI model, concomitant with a decrease in xanthine oxidase (XO) activity, XO-derived products (H_2_O_2_ and UA), and vascular peroxidase 1 expression [87]. In cellular experiments, the disruption of XO expression resulted in a reduction in apoptosis in cardiomyocytes subjected to hypoxia/reoxygenation (H/R) [87]. Importantly, XO, which is present in mammals, exhibits a molybdenum flavoprotein dimer structure and represents a variant of xanthine oxidoreductase (XOR). XO facilitates the conversion of hypoxanthine and xanthine into UA while simultaneously transferring electrons to molecular oxygen, resulting in the generation of ROS and reactive nitrogen species (RNS) such as peroxynitrite [88,89]. The etiology of HUA may be attributed to an upregulation of UA production caused by an increase in XO activity, accompanied by an elevation in ROS such as superoxide and H_2_O_2_. The prevailing oxidative redox state contributes to an amplified response to oxidative stress. Previous research has indicated that hyperoxic conditions inhibit XOR enzyme activity, whereas hypoxia promotes XOR expression [90] (Figure 4). This observation aligns with experimental findings demonstrating a larger area of cardiac infarction in the HUA group within the mouse model of MIRI [91]. Therefore, the activity of XOR plays a pivotal role in the ability of HUA to induce oxidative damage to cardiomyocytes, indicating that XOR is a promising therapeutic target for subsequent cardiac disorders.

#### 3.2.2. ROS-Mediated Activation of the NLRP3 Inflammasome

In the context of myocardial ischemia, the recirculation of blood triggers a process wherein oxygen reacts with dissolved substances originating from damaged or necrotic cardiomyocytes, thereby generating reactive species such as oxygen radicals. These reactive substances have the potential to cause oxidative harm to the myocardium (Figure 4). Shen et al. conducted both in vivo and in vitro experiments and revealed that UA exacerbates H/R in cardiomyocytes and MIRI in mice by stimulating the production of ROS and cellular pyroptosis [91]. Furthermore, it has been demonstrated that the employment of ROS scavengers and NLRP3 inhibitors effectively counteracts the aforementioned effect [91]. Additionally, Wu et al. [67] have reached the conclusion that UA induces myocardial infarction injury by activating the NLRP3 inflammasome and the ROS/TRPM2/Ca^2+^ pathway. Taken together, these findings consistently indicate that UA promotes the generation of ROS, leading to the activation of the NLRP3 inflammasome and exacerbating myocardial injury.

## 4. Uric acid-Lowering Interventions

The present clinical approach to managing HUA and gout primarily involves educational and outreach efforts, as well as pharmacological interventions. The most commonly prescribed urate-lowering medications include inhibitors of UA synthesis (such as allopurinol and febuxostat) and enhancers of UA excretion (such as benzbromarone). A cohort study, utilizing propensity score matching on a population-based sample, demonstrated that individuals with MI who received UA lowering therapy (ULT) exhibited a lower rate of all-cause mortality and a significantly reduced risk of recurrent MI compared to those who did not receive ULT [92]. The findings of a case-control study conducted in Spain demonstrated that allopurinol exhibited a protective effect against the risk of AMI. This protective effect was found to be strongly influenced by the duration of treatment and the level of SUA achieved by patients following treatment [93]. Additionally, animal studies have indicated that pretreatment with allopurinol mitigated myocardial injury in mice with HUA [63], and that treatment with benzbromarone in rats with HUA significantly decreased the occurrence of AF [61]. XOI exhibits potential organ-protective properties and has the ability to decrease SUA levels. A study conducted on nephrectomized rats with HUA demonstrated the protective effects of febuxostat against cardiac and renal injury. This study also emphasized the pathological importance of XO in cardiac and renal interactions. Additionally, the oral administration of XOI to mice effectively mitigated the toxic cardiac damage induced by Adriamycin [94,95]. The protective effects may be ascribed to the participation of XO in the purine metabolism process, leading to the formation of UA, while simultaneously generating detrimental substances like reactive oxygen free radicals. Nonetheless, the ALL-HEART study, a recent extensive prospective randomized trial investigating the use of allopurinol in individuals with ischemic heart disease, demonstrated no significant differences in the primary outcomes of nonfatal MI, nonfatal stroke, or cardiovascular mortality between patients randomly allocated to allopurinol and those receiving conventional therapy [96]. This outcome suggests that allopurinol is unlikely to improve cardiovascular outcomes.

The cardiovascular safety of febuxostat and allopurinol in patients diagnosed with gout has been a subject of controversy (Figure 4). This contentious issue is exemplified by the findings of two prominent trials, namely the CARES trial and the FAST trial. The CARES trial reported that febuxostat usage was linked to elevated rates of all-cause and cardiovascular mortality compared to allopurinol [97]. Conversely, the FAST trial demonstrated that prolonged febuxostat administration did not result in an augmented risk of mortality or serious adverse events [98]. In light of the contentious nature of the research, it remains imperative to direct our attention towards the underlying concerns surrounding the controversy and the cardiovascular safety of XOI. We need to clarify whether HUA is a causal factor in the risk of developing major cardiovascular events. And it is crucial to ascertain the veracity of the claim that prolonged ULT employing XOI augments the likelihood of cardiovascular ailments. Furthermore, it is worth exploring the feasibility of designating XOI as a standard secondary preventive therapeutic agent for cardiovascular disease. Lastly, it is pertinent to evaluate the necessity of administering XOI UA-lowering interventions to patients with asymptomatic HUA. Despite the 2020 American College of Rheumatology guidelines discouraging the initiation of ULT in individuals with asymptomatic HUA [99], recent research has demonstrated that asymptomatic HUA independently contributes to the risk of MI in adults [100] and increased mortality in elderly patients with CAD who undergo elective PCI [101]. Therefore, it is necessary to develop efficient new drugs for the prevention and treatment of cardiac comorbidities caused by HUA.

## 5. Summary and Outlook

The relationship between HUA and CVD has been a subject of growing interest in recent years. This review aims to provide a comprehensive review of the clinical and basic studies on the associations between HUA and AMI, AF, and HF over the past few decades. Additionally, we summarize and analyze the potential mechanisms underlying HUA-related myocardial injury. Under various pathological circumstances (including the direct impact of HUA on cardiomyocytes and the combination of HUA with MIRI), HUA may elicit inflammatory reactions by activating the NLRP3 inflammasome, disrupting energy metabolism in cardiomyocytes, influencing antioxidant defense mechanisms, and inducing the production of ROS to amplify the oxidative stress response. Consequently, these processes may result in myocardial damage and subsequent functional deterioration. Furthermore, we explore the importance of HUA in conjunction with myocardial injury managed through interventions aimed at reducing UA levels, while also posing inquiries regarding the potential cardiovascular risks associated with such interventions and the necessity of undergoing ULT for asymptomatic HUA. Regardless, the deleterious impact of HUA on human well-being represents a significant issue that warrants careful consideration and necessitates further investigation to facilitate the advancement of innovative preventive and therapeutic approaches for cardiovascular diseases linked to HUA.

## 6. Limitations

This narrative review lacked explicit inclusion/exclusion criteria for the search of medical databases. The studies included in this review were selected arbitrarily by the authors and may not provide a comprehensive representation of the topic discussed.
